# Federated Learning Authenticity Standard for Healthcare as derived from lessons in self-driving cars

**DOI:** 10.1038/s43856-026-01820-2

**Published:** 2026-07-29

**Authors:** Rui Santos, Pearse A. Keane

**Affiliations:** 1 Department of Ophthalmology, Stadtspital Zürich, Zürich, Switzerland; 2 Spross Research Institute, Zürich, Switzerland; 3 NIHR Biomedical Research Centre, Moorfields Eye Hospital NHS Foundation Trust and UCL Institute of Ophthalmology, London, UK

**Keywords:** Prognosis, Computational biology and bioinformatics

## Abstract

Santos et al. propose a six-level classification scale, modelled on the autonomous driving taxonomy, to distinguish simulation-based from operational federated learning studies in healthcare. Requiring authors to declare the achieved level in a single sentence within the abstract would make honest reporting of study design unavoidable.

## Teaser

In this Perspective, we highlight a critical mislabeling problem in healthcare federated learning research. Although federated learning is widely promoted as a privacy-preserving approach for multi-institutional artificial intelligence development, most published studies are still single-institution simulations that do not cross real organizational boundaries. As such, we propose a six-level classification scale (0 to 5), modeled on the autonomous driving taxonomy used in the automotive industry, that ranges from no federation through purely mathematical simulations to fully autonomous, continuously learning systems operating across separate institutions. We term it Federated Learning Authenticity Standard for Healthcare. Further we propose that authors declare in their studies the achieved level in a single sentence within the abstract, making this standard an honest description of study design unavoidable. We discuss the standard’s potential role as a reporting framework analogous to established guidelines for clinical trials and prediction model studies.

## Introduction: consequences of mislabeling

In 2016, Tesla introduced a feature called “Full Self-Driving.” The name was memorable, but the system still required a fully attentive driver and could not operate independently in all settings. Under the Society of Automotive Engineers (SAE) framework, this was a Level 2 system, not true autonomy^1^. The value of the SAE levels was not that they improved the technology, but that they gave manufacturers, regulators, and the public a shared language for describing what a system could really do. Federated learning in healthcare faces a similar taxonomy problem.

Federated learning (FL), introduced by McMahan and colleagues in 2017, is often presented as a way to train artificial intelligence (AI) models across institutions without moving sensitive patient data into a central repository^2^. That promise matters greatly in healthcare, where data sharing is often limited by legal, technical, and organizational barriers. But in practice, the label “federated learning” is often used for studies that differ greatly in how close they come to real multi-institutional deployment.

This matters significantly because most published healthcare FL studies are still simulations. A recent systematic review found that only 5.2% of studies involved real clinical application, while the vast majority were proofs of concept or simulated experiments on data that had already been brought together in one place^3^. A common approach is to split a centralized dataset into artificial subsets, train a model on each subset, and then combine the updates as if the data were held by different institutions. This can be useful for testing algorithms, but it is not the same as building a working multi-institutional FL system. It does not require cross-institutional governance, local deployment inside hospitals, or the technical work needed to train across real organizational boundaries. When all of this is described simply as “federated learning”, readers may overestimate how mature and deployable the technology really is^4^.

In this perspective, we argue that the field of healthcare federated learning suffers from a labeling problem, alike the initial taxonomy for self-driving cars, that obscures the gap between algorithm testing and operational deployment. We introduce the Federated Learning Authenticity Standard for Healthcare (FLASH), a six-level scale modeled on the Society of Automotive Engineers (SAE) driving automation levels, which distinguishes simulated from genuinely federated work. We propose FLASH as a minimal reporting standard - a single declarative sentence in every abstract - and discuss its implications for journals, funders, and health systems seeking to track genuine progress toward deployable, multi-institutional artificial intelligence.

## Lessons from self-driving cars: defining the FLASH taxonomy for federated learning

**Fig. 1:**
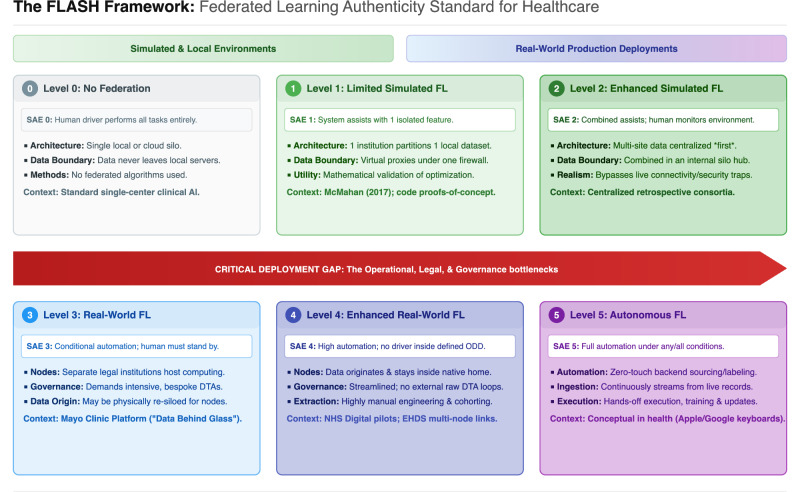
The Federated Learning Authenticity Standard for healthcare (FLASH) framework. The schematic shows the six Federated Learning Authenticity Standard for Healthcare levels arranged from least to most deployment-ready. Levels 0–2 represent studies in which no data cross real institutional boundaries, even when the underlying data originated at multiple sites. Levels 3–5 represent progressively more automated and operationally integrated multi-institutional systems. The central “Critical Deployment Gap” marks the transition from proof-of-concept work to genuinely operational federated learning. FL federated learning, SAE Society of Automotive Engineers, DTA data transfer agreement, EHDS European Health Data Space.

To address the current mislabeling which FL for healthcare suffers, we propose FLASH: the Federated Learning Authenticity Standard for Healthcare (Fig. 1 illustrates the framework, and Table 1 summarizes its main operational features). Like the SAE levels for driving automation, FLASH is designed to separate an attractive label from what a system is actually capable of doing in the real world^1^.

**Table 1:** Technical prerequisites and operational metrics of the Federated Learning Authenticity Standard for Healthcare taxonomy

FLASH level	Data topology	Governance threshold	Human intervention	Network infrastructure
**0: No Federation**	Single local repository	Internal IRB only	Full manual oversight	Local standalone compute
**1: Simulated FL**	Artificially partitioned single dataset	Single-institution DTA	Full manual oversight	Single localized server
**2: Enhanced Simulated**	Centralized, multi-site data	Multi-site DTA (centralized proxy)	Full manual oversight	Simulated network silos
**3: Real-World FL**	Geographically distinct nodes	Bespoke multi-entity DTAs	Manual pipeline initiation & curation	Cross-firewall, secure APIs
**4: Enhanced Real-World**	Geographically distinct nodes	Standardized data ontologies (OMOP/FHIR)	Manual initiation, automated aggregation	Standardized secure network
**5: Autonomous FL**	Live, distributed clinical streams	Automated/Smart DTAs	Zero-touch; continuous learning	Direct EHR/PACS integration

The table maps each of the six levels to its data topology, governance requirements, expected human intervention, and network infrastructure. Bold entries in the “Level” and “Data Topology” columns highlight the row label and the principal data-handling characteristic. “Simulated” refers to experiments that mimic multiple sites without training across real institutional boundaries; “real-world” refers to training performed across separate institutions that keep their data locally.

*FL* federated learning, *IRB* institutional review board, *DTA* data transfer agreement, *API* application programming interface, *OMOP* Observational Medical Outcomes Partnership, *FHIR* Fast Healthcare Interoperability Resources, *EHR* electronic health record, *PACS* picture archiving and communication system.

The framework FLASH has six levels. Level 0 denotes no federation: a model is trained within a single institution. Level 1 is simulated federation, in which a single dataset is artificially split to mimic multiple sites. Level 2 is enhanced simulated federation, in which data from multiple sites are first centralized and only then divided into virtual silos. The key boundary between simulation only and real world FL occurs between Levels 2 and 3. Up to Level 2, studies may test FL methods (namely for pipeline optimizations), but they do not yet demonstrate real-world federation. Level 3 is the first genuinely federated level where separate institutions keep their data locally and share only model updates, but the process still depends on substantial manual setup and governance. Level 4 adds more standardized infrastructure and automation, although humans still initiate and oversee the process. Level 5 comprises fully autonomous federated learning, in which local training and model updating occur continuously within live clinical systems.

A simple example illustrates the difference. At Level 1, researchers might take one centralized optical coherence tomography (OCT) dataset, split it into several parts, and train an AI model as if those parts came from different hospitals. At Level 2, they might use data originally collected at several sites but still bring everything together before simulating federation. By contrast, Level 3 begins only when each clinic keeps its own OCT data locally and participates in training across real institutional boundaries. Levels 4 and 5 then describe increasing degrees of standardization and automation. The point is not that lower levels are unimportant, because they are essential for algorithm testing and optimization, but that they should not be mistaken for real-world deployment.

FLASH is intended as a reporting framework. In practice, it could be stated in a single sentence in the abstract or methods of every FL study: “This study achieves FLASH Level X.” A paper based on one centralized dataset split into artificial nodes should not be presented as a real-world FL deployment, instead it should be described as FLASH Level 1. A study using data from several institutions that were first centralized should be described as FLASH Level 2. This small change in reporting would help reviewers, readers, and policymakers distinguish algorithm development from operational readiness much more quickly.

The main benefit of such a system is greater honesty about where the field stands. Journal adoption would be especially powerful, as reporting standards have already improved transparency in other areas of medical research, such as CONSORT and TRIPOD-AI^5,6^. It would also make two practical barriers more visible. The first is governance: even when raw data does not move, real-world FL usually still requires formal agreements between institutions. The second is infrastructure: higher FLASH levels depend on local computing resources, secure networking, and hospital IT support that many health systems do not yet have^4^. If studies state their FLASH level clearly, progress becomes easier to track, and claims become easier to interpret.

## Outlook

Adopting a single declarative standard is a deliberately modest proposal, but modest interventions can have large effects when they are widely accepted. The CONSORT standards for randomized trials and the more recent TRIPOD-AI statements^5,6^ for prediction models began as voluntary checklists and are now required by most major journals. The Federated Learning Authenticity Standard for Healthcare (FLASH) is proposed to follow a similar trajectory.

For such, three priorities follow. First, journals should be invited to require a Federated Learning Authenticity Standard for Healthcare (FLASH) level statement as a condition of submission for any paper that uses the term “federated learning”. This does not require a new peer review process, only a one-line check. Second, funders could use the standard to differentiate feasibility studies from operational deployment when allocating grants, and to track the proportion of funded work that crosses institutional boundaries. Third, health system information-technology leaders could use the standard to prioritize infrastructure investment as gaps between Federated Learning Authenticity Standard for Healthcare levels 2 and 3 are largely governance and network gaps, not algorithmic ones, and they are the most actionable by institutional information-technology leaders.

The automotive analogy is instructive in another way. The Society of Automotive Engineers levels did not stop manufacturers from using aspirational language, but they did make the language falsifiable. The Federated Learning Authenticity Standard for Healthcare offers healthcare federated learning the same falsifiability, and with it, a clearer measure of how far the field has come, and how far it still can go.

## Supplementary information

Transparent Peer Review file

## Data Availability

No data were used or generated in this study.
