# Supplementary material for: Federated Learning Authenticity Standard for Healthcare as derived from lessons in self-driving cars
Source: Commun Med (Lond). 2026 Jul 29;6:422. doi: 10.1038/s43856-026-01820-2 (PMC13427741; doi:10.1038/s43856-026-01820-2)
Supplement: Supplementary file 1 — Transparent Peer Review file [file 43856_2026_1820_MOESM1_ESM.pdf]

nature portfolio

Peer Review File

Federated Learning Authenticity Standard for Healthcare as derived from lessons in self-driving cars

Corresponding Author: Professor Pearse Keane

The manuscript was considered suitable for publication without review at Communications Medicine.

Open Access This Peer Review File is licensed under a Creative Commons Attribution 4.0 International License, which permits use, sharing, adaptation, distribution and reproduction in any medium or format, as long as you give appropriate credit to the original author(s) and the source, provide a link to the Creative Commons license, and indicate if changes were made.
In cases where reviewers are anonymous, credit should be given to 'Anonymous Referee' and the source.
The images or other third party material in this Peer Review File are included in the article’s Creative Commons license, unless indicated otherwise in a credit line to the material. If material is not included in the article’s Creative Commons license and your intended use is not permitted by statutory regulation or exceeds the permitted use, you will need to obtain permission directly from the copyright holder.
To view a copy of this license, visit https://creativecommons.org/licenses/by/4.0/
